# Isolated and Sequential Effects of Sodium Hypochlorite and Hydrogen Peroxide on Dentin Chemical Composition: An In Vitro FTIR and EDX Study

**DOI:** 10.3390/ma19132723

**Published:** 2026-06-25

**Authors:** María de las Gracias Ruiz, James Ghilotti, José Luis Sanz, Sofía Folguera, Carmen Llena

**Affiliations:** Department of Stomatology, Universitat de València, 46010 Valencia, Spain; mariadelasgraciasruiznavarro@gmail.com (M.d.l.G.R.); jose.l.sanz@uv.es (J.L.S.); sofia.folguera@uv.es (S.F.); llena@uv.es (C.L.)

**Keywords:** dentin, sodium hypochlorite, hydrogen peroxide, Fourier transform infrared spectroscopy, energy-dispersive X-ray spectroscopy

## Abstract

Sodium hypochlorite (NaOCl) remains the gold standard irrigant in endodontics due to its proteolytic and antimicrobial properties, whereas hydrogen peroxide (HP) is widely used for internal bleaching because of its oxidative capacity. Both agents have been associated with chemical and structural alterations in dentin; however, the impact of their sequential application on the organic–mineral balance has not been fully elucidated. Objective: To evaluate whether the isolated and sequential application of 5.25% NaOCl and 37.5% HP induces chemical alterations in dentin by analyzing changes in the organic matrix and mineral phase using Fourier-transform infrared spectroscopy (FTIR) and Energy-dispersive X-ray spectroscopy (EDX). Methods: Twenty-four independent dentin sections (n = 6 per group) from six human third molars were distributed using a tooth-balanced allocation into four groups: Control, NaOCl (5.25%, 15 min), HP (37.5%, 30 min), and sequential NaOCl+HP. FTIR assessed organic (amide I, II, III, CH_2_) and inorganic (phosphate, carbonate) components through baseline-corrected integrated areas, Full Width at Half Maximum (FWHM), and molecular ratios. Surface elemental composition and the calculated Ca/P atomic ratio were determined by EDX. Multiple sub-measurements per specimen were averaged before statistical analysis. Data were analyzed using Kruskal–Wallis and Mann–Whitney U tests with Bonferroni correction (*p* < 0.05). Results: FTIR revealed treatment-dependent modifications. NaOCl reduced absorbance in organic-associated bands, indicating collagen degradation, whereas HP altered the mineral phase. The NaOCl+HP group exhibited increased numerical values for integrated band areas, with differences detected in carbonate, phosphate, and amide III bands (*p* < 0.05), reflecting structural disorganization and modified spectral signal rather than tissue preservation. No differences were detected across the calculated infrared ratios (*p* > 0.05). EDX showed decreased absolute atomic percentages of Ca, P, and O in the NaOCl+HP group (*p* < 0.05), indicating structural demineralization, while its stoichiometric Ca/P ratio remained at 1.56. Isolated HP shifted the mineral stoichiometry to the highest numerical Ca/P ratio (1.69; range 1.58–1.80). Fluorine decreased across all treated groups (*p* < 0.001). Conclusions: Sequential NaOCl and HP application triggers distinct chemical alterations compared to individual treatments, inducing severe structural disorganization of the organic network and absolute mineral depletion of Ca and P. This multi-agent sequence alters dentin stoichiometry, which may compromise the biomechanical integrity of the tissue.

## 1. Introduction

The predictability of endodontic treatment relies on effective chemomechanical preparation and three-dimensional sealing of the root canal system. However, the inherent morphological complexity of root canals prevents mechanical instrumentation alone from achieving complete debridement, making chemical irrigation essential for reducing microbial load and removing the smear layer [1].

Sodium hypochlorite (NaOCl) remains the gold-standard irrigant due to its unique organic tissue-dissolving ability and broad antimicrobial spectrum [2,3,4,5,6,7]. Nevertheless, its clinical efficacy and its impact on the root canal wall are highly dependent on factors such as concentration, contact time, and temperature [8,9,10,11]. At higher concentrations or prolonged exposure, the chemical action of NaOCl can lead to collagen degradation, altered mechanical properties, and increased dentin porosity [12,13,14,15]. Therefore, understanding how NaOCl interacts with the dentinal substrate is crucial to balance its disinfection capacity with the preservation of the tooth’s structural integrity.

Following root canal treatment, endodontically treated teeth may require esthetic procedures such as internal bleaching. Hydrogen peroxide (HP) is one of the most used bleaching agents, typically applied in concentrations ranging from 5% to 40%. Its low molecular weight facilitates its penetration into dentin, where it acts as a strong oxidizing agent capable of breaking double bonds in both organic and inorganic compounds within dentinal tubules [16,17]. Compared to enamel, the effects of bleaching agents on dentin have been less extensively investigated. Previous studies have reported morphological changes in dentin surface, reductions in the calcium-to-phosphate ratio, and collagen degradation [18,19]. These alterations may compromise the mechanical properties of dentin, particularly at higher concentrations [20,21,22,23,24]. In addition, the generation of reactive oxygen species (ROS) during bleaching has been associated with the activation of matrix metalloproteinases (MMP-2 and MMP-9), promoting collagen degradation; this effect appears to depend on both the type and concentration of the peroxide used [16,19,25,26,27].

Based on the known deproteinizing effect of sodium hypochlorite and the demineralizing potential of hydrogen peroxide, and considering the limited evidence regarding their combined action on dentin, we hypothesized that the sequential use of NaOCl and HP would induce chemical alterations in dentin that differ from those produced by each agent alone.

Therefore, the aim of this study was to evaluate the chemical effects of the isolated and sequential application of 5.25% NaOCl and 37.5% HP on dentin by characterizing changes in both the organic matrix and the mineral phase using FTIR and EDX.

The null hypothesis was that the isolated or combined application of 5.25% NaOCl and 37.5% HP would not produce differences in the organic or mineral composition of dentin compared to the control group or among the experimental protocols.

## 2. Materials and Methods

The study was approved by the Human Research Ethics Committee of the Universitat de València (reference: 2023-ODON-3032285).

### 2.1. Sample Selection

A total of six impacted third molars were obtained from patients aged between 19 and 60 years after informed consent was obtained. The samples were examined under ×10 magnification using a stereomicroscope (Zeiss OPMI Lumera, Oberkochen, Germany) to confirm the absence of structural defects or damage. Subsequently, the teeth were stored in 0.2% thymol solution (Guinama, La Pobla de Vallbona, Valencia) until experimental processing.

### 2.2. Specimen Preparation

The teeth were initially embedded in acrylic resin (Resin Pro^®^, Resin Pro S.L., Barcelona, Spain) to form individual cylindrical blocks. Each specimen was then sectioned in a buccolingual direction to obtain dentin slices approximately 1 mm thick. A total of 24 dentin sections were obtained.

To ensure objectivity and prevent allocation bias, all dentin specimens were assigned a unique random alphanumeric code immediately after sectioning. Data acquisition for both FTIR and EDX, as well as the subsequent spectral and elemental analyses, were performed by operators who were blinded to the specific treatment allocation of the specimens. Furthermore, all spectroscopic measurements and integration windows were standardized and automated through software parameters, preventing investigator subjectivity from influencing data collection.

### 2.3. Experimental Groups

A total of 24 dentin sections were allocated into four groups using a tooth-balanced allocation (n = 6 per group). One untreated control group and three experimental groups. Due to the exploratory nature of the study and the technical complexity associated with specimen preparation and FTIR and EDX analyses, six specimens were included in each group. The untreated control group served as the baseline of reference for spectral and elemental characterization of dentin. All groups were analyzed under identical conditions and included in the statistical comparison. Accordingly, the experimental design was primarily focused on detecting treatment-related trends and chemical alterations among the experimental groups.

**Figure 1:**
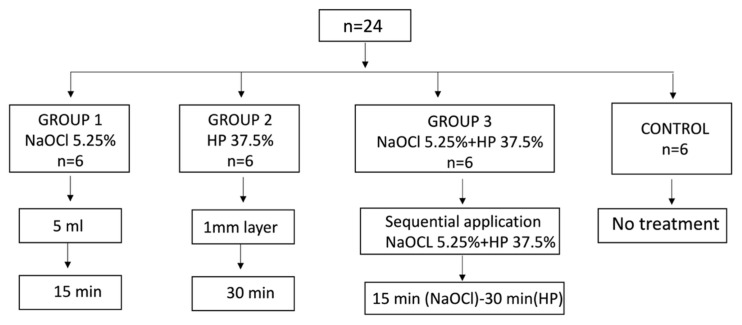
Distribution of experimental groups: Control (untreated baseline reference), NaOCl (sodium hypochlorite applied 15 min), HP (hydrogen peroxide applied 30 min), min: minutes. ml: milliliters.

In the experimental groups, specimens were treated as follows:

- Group 1 (NaOCl): immersion in 5.25% NaOCl (Dentaflux, J. Ripoll S.L., Algete, Madrid, Spain) for 15 min (5 mL).
- Group 2 (HP): application of a 1 mm-thick layer of 37.5% HP (Polaoffice, SDI, Victoria, Australia) for 30 min.
- Group 3 (NaOCl+HP): sequential application of the protocols described for Groups 1 and 2, with an intermediate rinse using phosphate-buffered saline (PBS) (PBS-A Labs, Vizcaya, Spain) (Figure 1).

After treatment, all samples were subjected to ultrasonic cleaning (3510 E-DTH, Branson, Danbury, CT, USA) in distilled water for 10 min and subsequently stored in PBS at 4 °C until analysis.

### 2.4. FTIR Analysis

**Figure 2:**
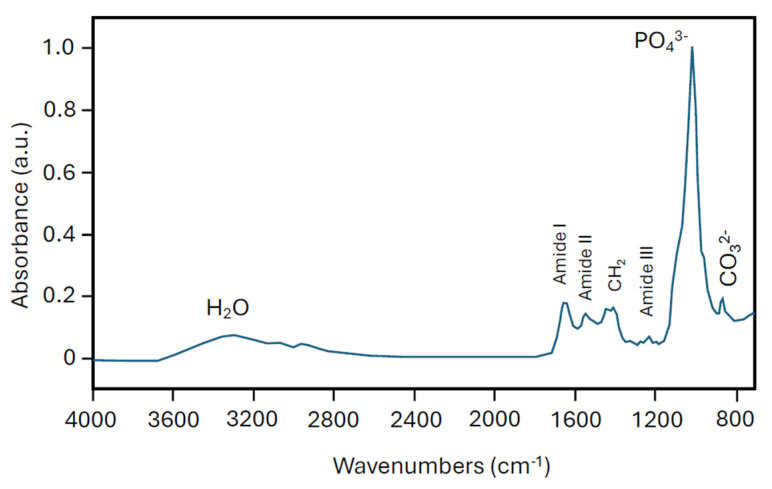
FTIR spectral profiles of human dentin sections across the experimental groups. Figure displays baseline-corrected spectra highlighting the organic-associated bands (Amide I, II, III, and CH_2_) and inorganic-associated bands (phosphate and carbonate). Experimental groups: Control (untreated baseline reference), NaOCl (5.25%, 15 min), HP (37.5%, 30 min), and sequential multi-agent treatment (NaOCl+HP). Wavenumbers are expressed in cm^−1^ on the *X*-axis, and Absorbtion intensity is displayed on the *Y*-axis.

FTIR was used to evaluate both the organic and inorganic components of dentin (Figure 2). Spectra were acquired at three different points on each specimen (central, mesial, and distal areas), performing 64 scans per point. Spectra were recorded within the range of 800–2000 cm^−1^ at a resolution of 4 cm^−1^, covering the characteristic regions of both the mineral (carbonate and phosphate) and organic components (CH_2_ and amide I, II, and III bands) [16,28,29]. To avoid pseudoreplication, the spectral data from the three evaluated locations (central, mesial, and distal) within the same specimen were averaged to produce a single, representative composite spectrum per independent experimental unit (n = 6 per group) prior to statistical analysis.

The bands corresponding to the inorganic component included carbonate (860–890 cm^−1^) and phosphate (950–1050 cm^−1^). For the organic component, CH_2_ (1410–1560 cm^−1^) and amide I (1600–1680 cm^−1^), amide II (1480–1580 cm^−1^), and amide III (1200–1300 cm^−1^) bands were analyzed. Spectral analysis was performed using a Cary 600 FTIR spectrometer (Agilent Technologies, Santa Clara, CA, USA).

The obtained spectral data were exported to Origin^®^ 7 software (OriginLab Corporation, Northampton, MA, USA) for processing. To eliminate baseline shifts and accurately isolate each baseline-corrected peak, a standard baseline subtraction was performed. To reduce instrumental noise without significantly altering spectral morphology, smoothing was performed using the Savitzky–Golay algorithm [30,31]. To address the inherent problem of band overlap in the complex infrared spectrum of dentin (particularly in the Amide I/II region and the Phosphate/Carbonate bands), baseline correction was standardly applied, and critical spectral regions were analyzed using fixed, well-defined integration windows for the area under the curve. This mathematical integration protocol ensured that minor peak shifting or shoulder overlaps did not compromise the relative quantification of the target functional groups [31,32].

The FWHM of the primary peaks was calculated as an indicator of the structural organization and crystallinity of dentin components. Additionally, quantitative band ratios (e.g., mineral-to-matrix ratio) were determined by dividing the integrated areas of selected peaks (such as phosphate/amide I) to establish quantitative relationships between the degradation or preservation of the organic matrix and the mineral phase of dentin [18,28,32].

### 2.5. EDX Analysis

To evaluate the mineral composition of dentin, a scanning electron microscope (SEM) (Hitachi S-4800, Hitachi High-Technologies Corporation, Tokyo, Japan) equipped with an EDX system (XFlash^®^ 5030, Bruker, Billerica, MA, USA) was used. Prior to analysis, specimens were sputter-coated with a gold–palladium alloy under an argon atmosphere using a Polaron Range SC7640 sputter coater (Quorum Technologies Ltd., Ashford, Kent, UK).

Acquisition parameters were set at a working distance of 10 mm, an accelerating voltage of 15 kV, a beam current of 100 μA, and an acquisition time of 100 s. The atomic weight percentage (%) of the main inorganic elements present in dentin was determined, including calcium (Ca), phosphorus (P), sodium (Na), magnesium (Mg), fluorine (F), and oxygen (O).

In each specimen, three measurement points were selected from the central, mesial, and distal areas of the dentin surface, avoiding edges and irregularities produced during specimen preparation. The final value assigned to each specimen corresponded to the arithmetic mean of the three measurements obtained.

### 2.6. Statistical Analysis

Data distribution was assessed using the Shapiro–Wilk test. As data were not normally distributed, non-parametric tests were applied. FWHM values for carbonate, phosphate, CH_2_, amide I, amide II, and amide III bands were expressed as median (minimum–maximum range). Comparisons among groups were performed using the Kruskal–Wallis test. The same analysis was applied to the phosphate/amide I, carbonate/phosphate, amide I/amide III, and CH_2_/amide I ratios. For the area-under-the-curve measurements, pairwise comparisons were performed using the Mann–Whitney U test. The elemental composition obtained by EDX analysis was also compared using the Mann–Whitney U test. To prevent Type I error inflation due to multiple pairwise evaluations, *p*-values were adjusted using the Bonferroni correction. Statistical significance was set at *p* < 0.05. Additionally, a post hoc power analysis was conducted based on the primary elemental outcomes (calcium and phosphorus atomic percentages obtained via EDX) to mathematically evaluate the adequacy of the sample size (n = 6). Considering the large effect sizes observed between the control and the multi-agent treated groups, the statistical power achieved was (1-beta) > 0.95, vastly exceeding the standard 80% threshold and confirming that the sample size was mathematically sufficient to reject the null hypothesis without incurring Type II errors.

## 3. Results

The results of the study will be presented in sections following the planning of the trials carried out.

### 3.1. FTIR Results

**Figure 3:**
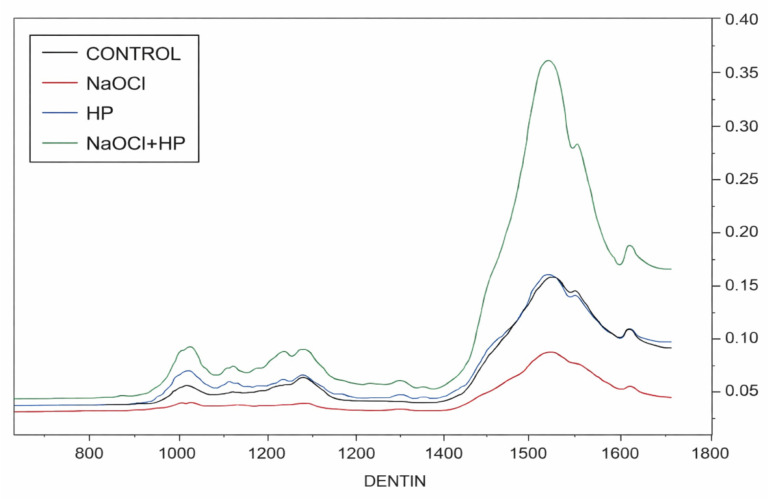
Superimposed FTIR absorbance spectra of human dentin across the experimental groups within the 700–1800 cm^−1^ region. The graph illustrates the treatment-dependent modifications in the molecular footprint of the substrate. The sequential multi-agent group (NaOCl+HP, green line) displays a marked numerical inflation and broadening across all bands (including carbonate, phosphate, and Amides), which typifies severe structural disorganization and phase alteration rather than component preservation. Conversely, the isolated NaOCl group (red line) shows a distinct drop in absorbance intensity, particularly within the organic-associated amide domains, reflecting its strong proteolytic collagen-dissolving capacity. The control (black line) and isolated HP (blue line) groups present intermediate absorbance profiles. Wavenumbers are displayed on the *X*-axis (cm^−1^), and baseline-corrected absorbance intensity is shown on the *Y*-axis (arbitrary units).

FTIR analysis allowed the characterization of both the organic and inorganic components of dentin in the control group and in the specimens subjected to the different treatment protocols. A comparative representation of the FTIR spectra is shown in Figure 3.

The FTIR spectral analysis revealed distinct absorbance profiles among the experimental groups across all evaluated bands. The NaOCl group exhibited decreased absorbance values, whereas the NaOCl+HP group displayed increased numerical values in both the mineral (carbonate and phosphate) and organic (CH_2_, amide I, II, and III) regions. This pattern potentially reflects structural disorganization and a relative modification of the spectral signal rather than the preservation of tissue components. The control and HP groups presented intermediate absorbance measurements across the evaluated spectral regions.

FWHM values were reported as median and minimum–maximum ranges. The Kruskal–Wallis test revealed no differences among the experimental groups for any of the analyzed variables (*p* > 0.05, Table 1). In the carbonate and amide III bands, the NaOCl and NaOCl+HP groups presented wider minimum–maximum ranges. For the phosphate band, the *p*-value for the NaOCl+HP group was *p* = 0.06.

Regarding the area under the curve, pairwise comparisons among experimental groups were performed using the Mann–Whitney U test (Table 2). The NaOCl+HP group exhibited increased integrated area values across all evaluated bands.

For the carbonate, phosphate, and amide III bands, the Mann–Whitney U test revealed differences between the NaOCl+HP group and both the control and the remaining experimental groups (*p* < 0.05). For the CH_2_, amide I, and amide II bands, the NaOCl+HP group presented larger numerical areas; however, no differences were detected when compared with the control group (*p* > 0.05), although differences were present in comparison with the NaOCl and HP groups (*p* < 0.05).

Table 3 presents the ratios calculated for the different experimental groups, expressed as median and minimum–maximum ranges. The Kruskal–Wallis test revealed no differences among groups for any of the evaluated ratios (*p* > 0.05).

For the phosphate/amide I ratio (*p* = 0.07), increased median values were detected in the NaOCl group and decreased values in the HP group, while the combined NaOCl+HP group presented values closer to the control group. Regarding the CH_2_/amide I ratio (*p* = 0.10), the NaOCl group presented the largest numerical median, whereas the HP and NaOCl+HP groups displayed lower numerical values. The amide I/amide III ratio (*p* = 0.31) presented similar measurements across all groups, with the lowest numerical median recorded in the NaOCl group. Finally, the carbonate/phosphate ratio (*p* = 0.18) showed comparable values among groups, with a numerical increase in the HP group and a decreased median in the combined group.

### 3.2. EDX Results

The predominant elements detected in all experimental groups were oxygen, calcium, and phosphorus, while sodium, magnesium, and fluorine presented lower numerical atomic percentages. Chlorine was below the detection limit in all groups (Table 4). Data represent the absolute atomic percentages (At%) of Calcium, Phosphorus, Oxygen, Sodium, Magnesium and Fluorine alongside the computed stoichiometric Ca/P ratio for each experimental group.

Statistical comparisons between groups were performed using the Mann–Whitney U test. The NaOCl+HP group exhibited a decrease in oxygen, calcium, and phosphorus atomic percentages compared with the control and the remaining experimental groups (*p* < 0.05). Sodium levels presented lower measurements in the NaOCl group than in the HP and the combined NaOCl+HP groups (*p* < 0.05). Regarding magnesium, increased values were recorded in the NaOCl and NaOCl+HP groups compared with the HP group (*p* < 0.05), with no differences detected in the remaining comparisons (*p* > 0.05). Fluorine was quantified across all groups, with the control group displaying higher atomic percentages than the treated groups (*p* < 0.001).

Regarding the elemental proportions, the calculated Ca/P ratio showed a distinct descriptive trend among groups. The HP group exhibited the highest Ca/P ratio (1.69, range 1.58–1.80), visibly shifting away from the Control group (1.49, range 1.47–1.51) due to the significant changes observed in individual Ca and P atomic percentages. Conversely, the ratios for NaOCl (1.56) and NaOCl+HP (1.56) remained closer to the baseline control values, suggesting that while these treatments alter the absolute mineral presence, they maintain a relatively stable calcium-to-phosphorus proportion in the remaining crystalline structure.

## 4. Discussion

The primary objective of this study was to evaluate the chemical, structural, and elemental alterations induced in the dentin matrix by the isolated and sequential application of 5.25% NaOCl and 37.5% HP. The main findings of this investigation demonstrate that while individual treatments selectively target specific dentin phases, their sequential combination (NaOCl+HP) triggers a synergistic degradation that drastically collapses both the organic matrix and the mineral phase. Specifically, FTIR analysis proved that NaOCl exerts a powerful deproteinizing effect, whereas HP alters the mineral crystallinity. Crucially, when used sequentially (NaOCl+HP), a profound loss of surface calcium and phosphorus occurs. These compositional variations alter the structural stoichiometry of the substrate, which directly rejects the null hypothesis.

The experimental protocol evaluated the effect of the isolated and sequential application of 5.25% NaOCl and 37.5% HP, with exposure times of 15 and 30 min, respectively. Previous evidence indicates that the action of these agents depends on their concentration, contact time, and sequence of application [12,33,34,35,36,37]. Regarding clinical relevance, a 5.25% NaOCl concentration represents the standard high-concentration irrigant utilized during endodontic therapy to maximize tissue dissolution and disinfection. Although in a clinical scenario NaOCl is continuously refreshed, a 15 min continuous exposure accurately mirrors the cumulative contact time of the irrigant inside the root canal system during a standard endodontic procedure [12,33]. On the other hand, a 37.5% HP concentration is widely used in clinical dentistry for in-office bleaching and intracoronal walking-bleach techniques in non-vital teeth. The selected 30 min exposure time aligns directly with the manufacturer’s recommendations and standard clinical protocols for professional bleaching, where a single application typically ranges between 20 and 45 min to achieve optimal chromophore oxidation without inducing safe-threshold cervical resorption [36,37]. Therefore, evaluating these specific time frames and concentrations allowed for a realistic simulation of the chemical stress that dentin undergoes during combined endodontic and internal bleaching therapies. Regarding the translational relevance of these findings, it must be acknowledged that while the selected concentrations and exposure times closely mirror standard clinical protocols, certain in vitro experimental conditions cannot fully replicate the dynamic environment of the oral cavity. In clinical practice, factors such as the positive outward pressure of dentinal fluid, the constant buffering capacity of surrounding tissues, and the progressive inactivation of NaOCl upon contact with organic matter may partially mitigate the severity of the chemical alterations observed in this study. Nevertheless, controlling these biological variables in our model was essential to isolate the direct impact of these agents on dentin chemistry. The severe structural disorganization and absolute mineral depletion detected in the sequential NaOCl+HP group highlight a critical chemical synergy that clinicians should carefully consider.

FTIR spectroscopy served to monitor functional group dynamics through FWHM and area under the curve calculations. A major challenge when analyzing dentin via FTIR is band overlap, where neighboring molecular vibrations (such as the overlapping profiles of Amides I, II, and III, or the proximity of carbonate shoulders to the main phosphate ν_1_, ν_3_ domain) can distort absolute intensity measurements. In this study, this limitation was strictly managed by evaluating integrated areas under baseline-corrected curves within fixed, standardized spectral windows rather than relying on raw peak heights. Consequently, the Phosphate/Amide I ratio acted as a reliable indicator of the mineral-organic balance, the carbonate/phosphate ratio monitored mineral phase substitutions, and the CH_2_/Amide I and Amide I/Amide III ratios tracked structural modifications within the collagenous framework [31,32,38].

In the FTIR analysis, the increase in the Phosphate/Amide I ratio in the NaOCl group reflects a relative reduction of the organic phase, driven by the well-known proteolytic capacity of NaOCl, which targets and breaks down the peptide chains of dentinal collagen [12,33,38,39]. This is further substantiated by the increased numerical median of the CH_2_/Amide I ratio (*p* = 0.10), which marks structural destabilization of the remaining organic matrix. Conversely, the decrease in the Phosphate/Amide I ratio (*p* = 0.07) observed in the isolated HP group points toward a preferential mineral depletion. HP acts as a potent oxidizing agent that modifies hydroxyapatite and carbonate networks without exhibiting strong proteolytic activity against collagen [37]. This aligns with the numerical increase observed in the carbonate/phosphate ratio (*p* = 0.18) under HP treatment, indicating an alteration in crystal organization and a potential shift toward carbonate substitution. When both agents were applied sequentially, the phosphate/Amide I ratio mathematically approached control values; however, this does not signify tissue preservation, but rather a parallel, destructive equilibrium where NaOCl destroys collagen while HP degrades the mineral phase simultaneously [37]. This structural disruption is supported by the Amide I/Amide III ratio (*p* = 0.31), where the drop in the numerical median for the NaOCl group indicates a loss of the native secondary conformation of collagen [40]. Furthermore, this interpretation is supported by the absolute integrated areas and FWHM trends of the individual bands. The sequential NaOCl+HP group exhibited an inflation of the Amide III and CH2 integrated areas, accompanied by a marked broadening of their FWHM values. In infrared spectroscopy, peak broadening without an increase in true material concentration is a classic hallmark of molecular chaos, crystallization loss, and phase transitions [40]. The sequential application of NaOCl and HP collapses the highly structured dentin scaffold; NaOCl strips away the protective collagen wrapper, fully exposing the underlying crystals to the aggressive oxidation of the 37.5% HP [37]. This dual chemical stress increases the rotational freedom of denatured matrix fragments and alters their spectral dipole moments, mathematically inflating the integrated area signals [40]. This structural disorganization is cross-validated by the EDX analysis, where a severe absolute depletion of Ca, P, and O directly refutes any hypothesis of tissue preservation or component accumulation. Potential alternative explanations like spectral artifacts or baseline shifts can be confidently ruled out, as all spectra underwent rigorous, standardized baseline subtraction and Savitzky–Golay smoothing, confirming that these spectral variations reflect true biophysical degradation rather than instrumental variance.

A deeper analysis of the inorganic phase was achieved through EDX elemental characterization, utilizing the calculated Ca/P atomic ratio (At%) as a direct indicators of hydroxyapatite stoichiometry [41,42]. Healthy human dentin presents a baseline Ca/P ratio typically ranging between 1.45 and 1.60 due to its carbonated, calcium-deficient nature. This is consistent with our Control group, which exhibited a stable ratio of 1.49 (range: 1.47–1.51). When evaluating the experimental treatments, the isolated HP group shifted the stoichiometry significantly, yielding the highest Ca/P ratio of 1.69 (range: 1.58–1.80). This specific outcome indicates that HP induces a preferential depletion of phosphate or a structural rearrangement that leaves a higher relative concentration of surface calcium atoms exposed, approaching the stoichiometric value of pure hydroxyapatite (1.67).

In contrast, the NaOCl group (1.56) and the sequential NaOCl+HP group (1.56) maintained a Ca/P ratio closer to the control line. Nevertheless, the EDX data reveals that their underlying chemical mechanisms are vastly different. In the NaOCl group, the absolute atomic percentages of Ca (23.70%) and P (15.20%) increased relative to the control. This phenomenon occurs because the dissolution of superficial collagen uncovers the underlying inorganic phase, rendering more crystals accessible to the EDX electron beam without altering the intrinsic Ca/P proportion.

Crucially, the sequential NaOCl+HP treatment resulted in a severe, highly significant drop in absolute Ca (15.40%) and P (9.90%) levels (*p* < 0.05). This massive elemental depletion represents the most critical finding of our study. The initial NaOCl rinse strips away the organic protection of the dentin tubules, leaving the mineral phase completely naked and vulnerable to the subsequent HP application, which violently destabilizes and demineralizes the exposed hydroxyapatite crystals. The wide range observed in the Ca/P ratio for this combined group (1.09–2.16) further highlights the severe and heterogeneous structural disintegration of the dentin surface. Clinically, a tooth substrate deprived of nearly half of its essential calcium and phosphate content is structurally compromised, implying a substantial risk of microhardness loss and increased fracture susceptibility under functional loading.

Regarding the minor elements, the increase in magnesium after isolated NaOCl treatment reflects the uncovering of the highly mineralized peritubular dentin following intertubular collagen removal [12,43]. The behavior of sodium also outlines distinct mechanisms: while NaOCl alone causes a leaching effect on the surface (*p* < 0.05) [44], the aggressive effervescence reaction generated during the sequential NaOCl+HP interaction promotes deeper penetration, creating a micro-porous surface that retains residual sodium ions within the damaged matrix [45,46]. Furthermore, the dramatic loss of fluoride across all treated groups (*p* < 0.001) serves as a definitive marker of superficial dentin erosion, as fluoride is highly concentrated in the outermost layers of the tissue [47]. Finally, the absence of residual chlorine confirms the effectiveness of the post-treatment rinsing protocol, ensuring that the observed effects are linked to the active chemical reactions rather than persistent reactive by-products [12,48].

This elemental depletion observed via EDX closely cross-validates the molecular shifts recorded in the FTIR analysis. The severe reduction in absolute calcium and phosphorus percentages explains the sharp increase in the integrated areas of the phosphate and carbonate bands in the sequential NaOCl+HP group. Rather than indicating an increase in mineral density, this phenomenon reflects a highly disorganized, porous, and carbonated surface where the collapsed collagen matrix leaves damaged hydroxyapatite crystal domains completely exposed to the infrared beam. Furthermore, the chaotic fluctuations in the EDX Ca/P ratio for the sequential group (ranging from 1.09 to 2.16) perfectly mirror the lack of statistical differences in the FTIR Phosphate/Amide I and Carbonate/Phosphate ratios (*p* > 0.05). This indicates that sequential multi-agent treatment does not induce a uniform, systematic demineralization, but instead triggers a severe, heterogeneous topographical dissolution where the stoichiometric equilibrium of the dentin surface is completely disrupted.

This study presents certain limitations that must be addressed. First, its in vitro design cannot replicate the dynamic clinical environment, such as the positive pressure of dentinal fluid or systemic temperature regulation. Second, EDX operates as a surface characterization tool, measuring elemental shifts within the first few microns, which may not represent deeper radicular dentin. Third, despite managing band overlap in FTIR through baseline and integration strategies, spectral constraints remain an inherent limitation of infrared analyses.

Fourth, a limitation involves the use of PBS as the storage solution. While PBS is widely accepted to maintain osmotic pressure and prevent tissue dehydration [33,49], its high phosphate content carries a risk of superficial mineral deposition or ion exchange with calcium-deficient dentin during prolonged storage. Although all specimens in this study were stored under identical conditions to ensure internal validity, this buffering effect could slightly mask the absolute extent of demineralization caused by the active agents.

A limitation of this study relates to the sample size. Although six specimens were included per group, which may limit the generalizability of the findings, this number is consistent with previous exploratory biomaterial studies employing FTIR and EDX analyses to investigate dentin chemical alterations [32,33,38]. In addition, each experimental group included one dentin section obtained from each of the six teeth used in the study, ensuring a balanced distribution of tooth-related biological variability across all treatment groups. The post hoc power analysis confirmed a statistical power exceeding 95% for the primary elemental outcomes, supporting the adequacy of the sample size for detecting the observed effects. Furthermore, no direct mechanical assessments (such as nanoindentation or fracture toughness tests) were performed, preventing a direct numerical correlation between the chemical alterations observed and the resulting biomechanical behavior of dentin. Future studies incorporating larger sample sizes, mechanical testing, and long-term structural evaluation are warranted. Nevertheless, the concordant findings obtained by FTIR and EDX provide robust mechanistic evidence that the sequential application of NaOCl and HP induces distinct alterations in both the organic and inorganic components of dentin.

## 5. Conclusions

With the inherent limitations of in vitro studies, based on the results obtained, it can be concluded that the combined treatment (NaOCl+HP) produced the greatest impact on dentin, both structurally and chemically.

FTIR analysis revealed that all treatments affected Amide bands (I, II, and III) and CH_2_ groups. The NaOCl group showed the lowest band intensities, confirming its strong proteolytic capacity to dissolve collagen. However, despite absolute variations in mineral and protein content, the ratios (phosphate/Amide I and carbonate/phosphate) did not show statistically significant differences. This suggests that, although there is substantial substrate loss (especially with NaOCl+HP), the relative proportion of the remaining components within the crystalline network remains relatively stable.

EDX analysis confirmed a critical and significant reduction in calcium and phosphate levels in the NaOCl+HP group, indicating severe demineralization that establishes a chemical substrate highly susceptible to biomechanical impairment more than any individual treatment.

Further studies are required to correlate these chemical alterations with mechanical properties under clinically relevant conditions.

## Figures and Tables

**Table 1:** Integrated area values of the organic and inorganic infrared bands of human dentin across the experimental groups. Data represents FWHM reported as medians and interquartile range (Q1–Q3).

	Carbonate (*p* = 0.47)	Phosphate (*p* = 0.08)	CH_2_ (*p* = 0.16)	Amide I (*p* = 0.74)	Amide II (*p* = 0.20)	Amide III (*p* = 0.16)
Control	88.42 (77.75–94.39)	134.91 (130.91–135.32)	156.83 (151.91–231.96)	87.61 (84.37–87.34)	144.20 (142.95–144.44)	131.32 (130.27.204.66)
NaOCl	129.64 (112.69–130.15)	130.44 (130.22–133.58)	216.09 (197.22–220.22)	105.72 (93.16–117.45)	142.13 (138.62–189.62)	272.72 (226.62–344.64)
HP	82.51 (79.58–109.39)	134.36 (131.93–139.22)	279.15 (259.27–311.74)	82.85 (82.33–84.23)	133.80 (129.00–134.01)	229.16 (217.93–251.53)
NaOCl+HP	100.32 (98.17–112.46)	115.93 (114.63–117.27)	287.04 (274.29–328.01)	81.38 (80.34–85.40)	148.61 (127.34–151.42)	320.20 (308.78–348.77)

NaOCl: Sodium hypochlorite (5.25%, 15 min); HP: Hydrogen peroxide (37.5%, 30 min); NaOCl+HP: Sequential multi-agent application.

**Table 2:** Area under the curve of FTIR values of the major mineral and organic infrared bands of human dentin. values reflect the structural crystallinity of the mineral phase (phosphate and carbonate bands) and the conformational order of the organic collagenous matrix (Amide I band). Values are expressed in wavenumbers (cm^−1^) as medians and interquartile range (Q1–Q3).

	Carbonate	Phosphate	CH_2_	Amide I	Amide II	Amide III
Control	2.43 ^a^ (1.97–3.17)	12.35 ^a^ (10–59–16.45)	4.17 (3.42–5.93)	2.16 (1.76–3.54)	2.14 (1.76–3.34)	1.35 ^a^ (1.11–1.94)
NaOCl	0.85 ^b^ (0.79–0.92)	4.26 ^b^ (3.97–4.32)	1.18 ^a^ (1.03–1.26)	0.57 ^a^ (0.53–0.60)	0.64 ^a^ (0.57–0.71)	0.38 ^b^ (0.35–0.52)
HP	1.52 ^c^ (1.25–1.73)	5.54 ^c^ (5.17–6.88)	2.72 ^b^ (2.68–3.83)	1.62^b^ (1.56–2.44)	1.63 ^b^ (1.59–2.31)	1.13 ^c^ (1.05–1.44)
NaOCl+HP	4.57 ^a,b,c^ (4.25–4.74)	27.19 ^a,b,c^ (24.77–28.92)	7.84 ^a,b^ (7.41–7.84)	4.95 ^a,b^ (4.73–5.33)	4.55 ^a,b^ (4.50–4.63)	2.85 ^a,b,c^ (2.82–2.92)

NaOCl: Sodium hypochlorite (5.25%, 15 min); HP: Hydrogen peroxide (37.5%, 30 min); NaOCl+HP: Sequential multi-agent application. Different superscript letters within the same column denote statistically significant pairwise differences (*p* < 0.05) based on Mann–Whitney U post hoc analysis with Bonferroni adjustment.

**Table 3:** Relative molecular and stoichiometric ratios of mineral-to-organic and inorganic components in human dentin. The table displays the calculated relative ratios derived from integrated FTIR band areas, including the phosphate/Amide I ratio (mineral/organic baseline balance) and the carbonate/phosphate ratio (crystalline substitutions). Values are expressed as medians and interquartile range (Q1–Q3).

	Phosphate/Amide I (*p* = 0.07)	Carbonate/Phosphate (*p* = 0.18)	Amide I/Amide III (*p* = 0.31)	CH_2_/Amide I (*p* = 0.10)
Control	6.10 (5.03–7.45)	0.197(0.14–0.19)	1.60 (1.59–1.63)	1.93 (1.57–1.97)
NaOCl	6.21 (5.57–6.93)	0.20 (0.17–0.25)	1.00 (0.89–1.12)	2.07 (1.76–2.13)
HP	3.46 (3.32–3.56)	0.28 (0.20–0.34)	1.49 (1.46–1.50)	1.68 (1.51–1.75)
NaOCl+HP	2.41 (2.26–2.67)	0.17 (0.15–0.40)	1.68 (1.67–1.71)	1.58 (1.22–1.74)

NaOCl: Sodium hypochlorite (5.25%, 15 min); HP: Hydrogen peroxide (37.5%, 30 min); NaOCl+HP: Sequential multi-agent application.

**Table 4:** EDX elemental analysis and calculated stoichiometry of dentin. Values are expressed as medians and interquartile range (Q1–Q3).

	Oxygen	Calcium	Phosphorus	Sodium	Magnesium	Fluorine	Ca/P ratio
Control	61.60 ^a^ (61.51–62.00)	21.30 ^a^ (21.15–21.40)	14.30 ^a^ (14.20–14.30)	1.70 (1.70–1.75)	0.70 (0.65–0.75)	0.20 ^a,b,c^ (0.20–0.40)	1.49 ^a^ (1.47–1.51)
NaOCl	60.67 ^b^ (59.94–62.14)	25.19 ^b^ (24.58–25.35)	14.24 ^b^ (14.12–14.46)	1.08 ^a,b^ (1.03–1.13)	0.59 ^a^ (0.56–0.61)	0 ^a^ (0–0.01)	1.76 ^a^ (1.46–1.68)
HP	59.74 ^c^ (59.19–60.22)	24.59 ^c^ (24.28–24.98)	13.96 ^c^ (13.56–14.13)	1.18 ^a^ (1.03–1.30)	0.58 ^a,b^ (0.51–0.60)	0.01 ^b^ (0–0.03)	1.76 (1.58–1.80)
NaOCl+HP	56.28 ^a,b,c^ (54.08–58.04)	19.02 ^a,b,c^ (17.72–19.42)	9.90 ^a,b,c^ (9.50–11.20)	1.36 ^b^ (1.18–1.51)	0.65 ^b^ (0.61–0.71)	0.01 ^c^ (0–0.02)	1.92 ^a^ (1.10–2.16)

NaOCl: Sodium hypochlorite (5.25%, 15 min); HP: Hydrogen peroxide (37.5%, 30 min); NaOCl+HP: Sequential multi-agent application. Different superscript letters within the same row denote statistically significant pairwise differences (*p* < 0.05) calculated via Mann–Whitney U tests with Bonferroni correction for multiple comparisons.

## Data Availability

The original contributions presented in this study are included in the article. Further inquiries can be directed to the corresponding author.

## Abbreviations

The following abbreviations are used in this manuscript:

NaOCl	Sodium hypochlorite
HP	Hydrogen peroxide
FTIR	Fourier-transform infrared spectroscopy
EDX	Energy-dispersive X-ray spectroscopy
